# Sex Specific Differences in Fetal Middle Cerebral Artery and Umbilical Venous Doppler

**DOI:** 10.1371/journal.pone.0056933

**Published:** 2013-02-20

**Authors:** Tomas Prior, Marianne Wild, Edward Mullins, Phillip Bennett, Sailesh Kumar

**Affiliations:** 1 Centre for Fetal Care, Queen Charlotte's and Chelsea Hospital, London, United Kingdom; 2 Institute for Reproductive and Developmental Biology, Imperial College London, London, United Kingdom; Université de Montréal, Canada

## Abstract

**Background:**

The incidence of several adverse pregnancy outcomes including fetal growth restriction are higher in pregnancies where the fetus is male, leading to suggestions that placental insufficiency is more common in these fetuses. Placental insufficiency associated with fetal growth restriction may be identified by multi-vessel Doppler assessment, but little evidence exists regarding sex specific differences in these Doppler indices or placental function. This study aims to investigate sex specific differences in fetal and placental perfusion and to correlate these changes with intra-partum outcome.

**Methods and Findings:**

This is a prospective cohort study. We measured Doppler indices of 388 term pregnancies immediately prior to the onset of active labour (≤3 cm dilatation). Fetal sex was unknown at the time of the ultrasound assessment. Information from the ultrasound scan was not made available to clinical staff. Case notes and electronic records were reviewed following delivery. We report significantly lower Middle Cerebral artery pulsatility index (1.34 vs. 1.43, p = 0.004), Middle Cerebral artery peak velocity (53.47 cm/s vs. 58.10 cm/s, p = <0.001), and Umbilical venous flow/kg (56 ml/min/kg vs. 61 ml/min/kg, p = 0.02) in male fetuses. These differences however, were not associated with significant differences in intra-partum outcome.

**Conclusion:**

Sex specific differences in feto-placental perfusion indices exist. Whilst the physiological relevance of these is currently unknown, the identification of these differences adds to our knowledge of the physiology of male and female fetuses in utero. A number of disease processes have now been shown to have an association with changes in fetal haemodynamics in-utero, as well as having a sex bias, making further investigation of the sex specific differences present during fetal life important. Whilst the clinical application of these findings is currently limited, the results from this study do provide further insight into the gender specific circulatory differences present in the fetal period.

## Introduction

The ratio of male fetuses to female fetuses is known to differ at conception [1], at miscarriage [2], and for stillbirths [3], with male fetuses more likely to be associated with adverse pregnancy outcomes. Previous studies have also demonstrated increased rates of intra-partum fetal distress [4] and Caesarean section for male fetuses [5]. Disorders of pregnancy associated with poor placentation such as abruption [6], pre-eclampsia [7] and fetal growth restriction associated with placental insufficiency [8] have also been shown to occur more frequently when the fetus is male. These observations have led to suggestions that there is a gender specific association with poor placentation and that placental insufficiency is more common in pregnancies with a male fetus than those with a female fetus.

The development of techniques to identify placental insufficiency has focused on growth restricted fetuses. Circulatory adaptations in these fetuses include evidence of increased placental resistance demonstrated by an increase in Umbilical artery resistance indices [9]. Imaging of the fetal cranial circulation in cases of growth restriction has identified evidence of cerebral redistribution [10]. This adaptation preserves blood supply to the brain, and may be identified by a reduction in Middle Cerebral artery resistance indices [11], [12]. While cerebral redistribution is considered protective in utero, some studies suggest that the development of this “brain sparing” circulation may be associated with adverse long term neurological outcomes [13]. Studies of Umbilical venous flow have demonstrated reduced venous flow rates in growth restricted fetuses [14].

Whilst male fetuses have been demonstrated to be at increased risk of various obstetric complications related to placental function, there has been little evaluation and comparison of Doppler indices suggestive of placental dysfunction between male and female fetuses.

The aim of this study was to ascertain the differences in Umbilical artery, Middle cerebral artery, Uterine artery, and Umbilical vein flow velocity waveforms between male and female fetuses, when measured prior to the onset of active labour (≤3 cm dilatation). Sex specific differences in Doppler resistance indices and velocimetry could indicate essential differences in placental perfusion and function between male and female fetuses.

## Methods

Three hundred and eighty eight women booked at Queen Charlotte's and Chelsea Hospital, Imperial College Healthcare Trust, London, W12 0HS, were recruited to this prospective cohort study over a 1 year period between January 2011 and January 2012. Women with uncomplicated term pregnancies were recruited to enter the study. Exclusion criteria included multiple pregnancy, known placental pathology such as fetal growth restriction/pre-eclampsia/pregnancy induced hypertension, known fetal anomaly, cervical dilatation ≥4 cm, and maternal age <16 years. Ethical approval for this study was granted by the North London Research Ethics committee (Ref No. REC 10/H0718/26). All participants recruited to the study gave informed written consent for their inclusion.

Each woman recruited to the study underwent an ultrasound assessment just prior to labour (GE Voluson e, 4–8 MHz trans-abdominal transducer) for measurement of fetal biometry, Umbilical, Middle Cerebral, and Uterine artery resistance indices, Umbilical venous flow velocity and vessel diameter, and amniotic fluid volume. Ultrasound examinations were performed with women lying supine, with the head of the bed elevated at a 45 degree angle. For all Doppler parameters, recordings were taken in the absence of fetal breathing movements. An automated tracing method was used incorporating at least 3 waveforms, and repeated 3 times in order to attain a mean pulsatility index and flow velocity. The angle of insonation of the vessel was kept <30 degrees. The Middle Cerebral artery was imaged using colour Doppler. Velocity waveforms were recorded from the proximal third of the vessel, distal to its origin at the circle of Willis. Umbilical artery and venous Doppler waveforms were recorded from a free loop of cord. The Umbilical vein was also imaged in grey scale, at a free loop, in longitudinal section, and the internal diameter recorded. The fetal sex was not ascertained at the time of the ultrasound assessment. All scans were performed by a single trained practitioner who had two years of advanced ultrasound training including performing multi-vessel fetal Dopplers. Each parameter was measured three times and the mean of these values used for data analysis. Labour was then managed according to local protocols and guidelines. Information from the ultrasound scan was not made available to the obstetric staff managing the labour. Following delivery, intra-partum and neonatal outcome details were recorded from the case notes and electronic patient records.

The cohorts of male and female fetuses were compared using independent sample t-tests (Mean Umbilical artery pulsatility index (UA PI), Middle Cerebral Artery pulsatility index and peak systolic velocity (MCA PI/PSV), Uterine artery PI, cerebro-umbilical and cerebro-uterine ratios, Umbilical venous flow rate, birth weight, gestation matched birth weight centile) and Chi squared tests (incidence of Caesarean section for fetal distress). For all statistical tests the significance level was set at p≤0.05. A diagnosis of fetal distress was based on either CTG abnormalities classified as pathological by the National Institute for Health and Clinical Excellence (NICE) guidelines [15] (variability <5 bpm for >90 minutes, persistent atypical/late decelerations, persistent bradycardia >3 minutes) and/or evidence of fetal acidaemia on fetal blood sampling (pH<7.20).

### Ethics Statement

Ethical approval for this study was granted by the North London Research Ethics committee (Ref No. REC 10/H0718/26). All participants recruited to the study gave informed written consent for their inclusion.

## Results

Maternal demographics are listed in Table 1. Three hundred and eighty eight women were recruited to the study over a one year time period. Of the 388 babies delivered 212 (54.6%) were male and 176 (45.4%) were female. The mean gestation at ultrasound was 40 weeks and 2 days (range = 37–42 weeks), with a mean interval between ultrasound and delivery of 29 hours). There was no significant difference in gestation between male and female infants (40+2 and 40+3 weeks, p = 0.08) The overall mean birth weight was 3520 g (range 1780 g–4940 g). The mean birth weight for the entire cohort was on the 53^rd^ centile. Male infants had a mean birthweight of 3563 g (range 1780 g–4940 g) which was on the 56^th^ centile and female infants had a mean birthweight of 3469 g (range 2144 g–4858 g) which was on the 49^th^ centile. The difference in mean birth weight and mean gestation matched birth weight centile was statistically significant (p = 0.049 and 0.018 respectively).

**Table 1:** Maternal Demographics.

Patient Demographics				
	Overall	Male Cohort	Female Cohort	p =
**Number of Patients**	388	212	176	n/a
**Maternal Age**	32 (20–47)	32	32	0.45
**BMI**	24 (17–42)	24	25	0.12
**% Primiparous**	65% (251/388)	64% (136/212)	65% (115/176)	0.86
**Ethnicity (%)**				
**Caucasian**	67% (260/388)	68% (144/212)	66% (116/176)	0.84
**Asian**	17% (66/388)	19% (40/212)	15% (26/176)	0.31
**Afro-Caribbean**	10% (38/388)	10% (21/212)	10% (17/176)	1
**Other**	6% (23/388)	3% (6/212)	10%(17/176)	0.002

Umbilical artery and Middle cerebral artery Doppler indices were recorded from all fetuses. The mean UA PI, MCA PI, and MCA peak systolic velocity (PSV) for the entire cohort were 0.80 (range 0.45–1.53), 1.38 (range 0.73–2.29), and 55.56 cm/s (range 19.52 cm/s–115.61 cm/s) respectively. No significant difference was observed between the UA PI in male and female infants (0.80 vs. 0.80, p = 0.81). Male infants had a significantly lower mean MCA PI compared to female infants (1.34 vs. 1.42, p = 0.004) as well as significantly lower MCA peak systolic velocity (53.47 cm/s vs. 58.10 cm/s, p = <0.001). Forty four infants were delivered by emergency Caesarean section for fetal distress. Of these, 27 were male (61%) and 17 female (39%). When compared to the sex ratio of our study population (54.6% male, 45.4% female) the increased incidence of Caesarean section for fetal distress in male fetuses was not statistically significant (p = 0.36).

Umbilical venous flow was calculated (assuming laminar flow in a circular vessel) using the formula:
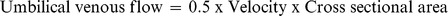



No significant difference in Umbilical vein diameter was found between male and female fetuses (7.21 mm vs. 7.22 mm, p = 0.85). Umbilical venous flow velocity was observed to be significantly lower in male fetuses (16.08 cm/s vs. 16.82 cm/s, p = 0.009). Male fetuses also had significantly lower umbilical venous flow per kilogram birth weight (56.78 ml/min/kg vs. 60.65 ml/min/kg, p = 0.02). These Doppler values are summarized in Table 2.

**Table 2:** Doppler Ultrasound values.

Doppler Values				
	Overall	Male Cohort	Female Cohort	p =
**Umbilical artery PI**	0.80	0.80	0.80	0.81
**Middle Cerebral artery PI**	1.38	1.34	1.42	0.004
**Middle Cerebral artery PSV**	55.56	53.47	58.10	<0.001
**Uterine Artery PI**	0.75	0.75	0.74	0.69
**Cerebro-umbilical ratio**	1.77	1.74	1.81	0.10
**Cerebro-uterine ratio**	2.01	1.95	2.10	0.09
**Umbilical venous flow velocity (cm/s)**	16.42	16.08	16.82	0.009
**Umbilical venous flow/kg (ml/kg/min)**	58.53	56.78	60.65	0.02

Uterine artery flow velocity waveforms were recorded in 74.7% (290/388 cases, 164/212 males and 126/176 females) of cases. It was not possible to adequately image the uterine vessels in the remaining cases due to the maternal habitus and late gestation. The mean Uterine artery pulsatility index was 0.75 (0.68–2.14). No significant difference in Uterine artery PI was observed between male and female fetuses (0.75 vs. 0.74, p = 0.69).

The cerebro-umbilical (MCA PI/UA PI) and cerebro-uterine (MCA PI/Uterine artery PI) ratios were also calculated. Both the cerebro-umbilical are cerebro-uterine ratios were found to be higher in female fetuses (1.74 vs. 1.81 and 1.95 vs. 2.1 respectively) but this difference did not reach statistical significance (p = 0.10 and 0.09 respectively).

Amniotic fluid volume was also compared between male and female infants and no statistically significant difference was observed in either the Amniotic Fluid Index (9.1 vs. 9.2, p = 0.78), or deepest vertical pool (4.1 vs. 4.3, p = 0.71).

As the study cohort contained several infants with birth weights <10^th^ centile (n = 26), who may have been growth restricted, we repeated our data analysis excluding these small for gestational age babies. Whilst the difference in mean birth weight and mean gestation matched birth weight centile was no longer statistically significant (3612 g vs. 3545 g, p = 0.13, and 59^th^ vs. 53th centile, p = 0.07), the difference in MCA PI, MCA PSV, and Umbilical venous flow/kg between male and female infants remained significant (1.34 vs. 1.43, p = 0.004, 53.71 cm/s vs. 58.24 cm/s, p = <0.001 and 57 vs. 61 ml/min/kg, p = 0.03).

## Discussion

In this study we report evidence of relatively reduced MCA resistance, MCA PSV and umbilical venous flow rates in male fetuses when compared to females. Sex specific variations in these Doppler indices have not been previously reported. We observed statistically significant differences in the MCA PI, MCA PSV, Umbilical venous flow velocity, and Umbilical venous flow/kg. These differences remained statistically significant even when infants considered small for gestational age (birth weight <10^th^ centile) were excluded from the analysis. No sex specific differences were observed in Umbilical artery PI, Uterine artery PI, the cerebro-umbilical or cerebro-uterine ratios.

Previous authors have suggested male fetuses are more likely to have placental insufficiency. This may be responsible for the increased rates of spontaneous miscarriage, in utero fetal demise, fetal growth restriction, and fetal distress in labour seen in pregnancies with a male fetus [16]. Edwards et al (2000) reported higher rates of severe placental dysfunction in male fetuses, evidenced by absent or reversed end diastolic flow in the umbilical artery in a cohort of growth restricted fetuses [8]. Our results suggest that subtle differences in the flow characteristics of the Middle cerebral artery exist between male and female fetuses, even in the presence of apparently normal placental function (as evidence by appropriate growth, normal umbilical and uterine artery Doppler resistance indices). Increased cerebral blood flow is a protective mechanism employed by the fetus during periods of hypoxia and is associated with fetal growth restriction [17]. The observation of a reduction in Middle Cerebral artery resistance in male fetuses when compared to female counterparts may represent a subtle adaptation to differing levels of placental function. Cerebral redistribution in small for gestational age fetuses has been shown to result in a higher risk of subsequent neuro-developmental deficits at two years of age [18]. Several neuro-developmental deficits such as language delay [19] and autistic spectrum disorders [20] are also known to have a male sex bias. Whilst data from the current study is purely observational, it does warrant further investigation of sex-specific changes in fetal haemodynamics. The differences between male and female fetuses observed here were subtle, but our study population included only appropriately grown foetuses. The differences in cerebral perfusion between the sexes may be more pronounced in cases of placental dysfunction, and could in part, explain the higher risk of poor neuro-developmental outcomes seen in males in pregnancies complicated by fetal growth restriction.

The cerebro-umbilical ratio has been suggested as the most accurate method of identifying a pathological brain sparing circulation [21] in growth restricted fetuses. However, we did not observe a significant difference in the cerebro-umbilical ratio between male and female fetuses. This is unsurprising considering this study focused on appropriately grown infants, unlikely to have significantly perturbed placental function.

Our results also demonstrate a reduced Umbilical venous flow rate in male fetuses when compared to female fetuses, in the absence of a difference in Umbilical artery Doppler resistance indices. The difference in flow rate was due only to reduced flow velocities in male fetuses, as there was no difference in the size of the Umbilical vein between the different sexes. Other authors have reported reduced Umbilical venous flow velocity in cases of fetal growth restriction and speculated that these changes occur prior to those in the Umbilical artery [22]. Our results suggest lower umbilical venous flow rates/kg for term fetuses than were previously reported by Rigano et al [14]. This discrepancy is due to the use of different formulae to calculate flow in a vessel. When calculating flow within a cylindrical vessel it is important to adjust for the differing flow rates within that vessel, with flow at the centre of the vessel occurring at a greater velocity than flow at its perimeter (due to friction with the vessel walls). This is corrected for by the multiplication factor of 0.5 in our formula. The paper by Rigano et al does not include this adjustment. Without this adjustment our figures for umbilical venous flow/kg are very similar to those previously published. Our results demonstrate a reduction in umbilical venous flow rate in male infants, without evidence of a similar change in the Umbilical artery.

Sex specific differences have also been observed in the fetal response to a sub-optimal intra-uterine environment. In pregnancies complicated by untreated maternal Asthma, female fetuses have reduced birth weight, whereas male fetal birth weight is unaffected [23]. Furthermore, sex specific alterations in placental genes involved with growth and inflammation have also been observed in cases of maternal hypoxia suggesting that aberrations in placental functions can occur in a sex specific manner [24]. The identification of sex specific differences in Middle Cerebral artery and Umbilical venous flow characteristics in this study necessitates further investigation to establish the physiological significance of this finding.

All ultrasound scans in this study were performed by a single trained clinician, limiting inter-observer variability. The fetal sex was unknown at the time of the ultrasound, thus limiting bias. Potential confounding factors such as ethnicity, maternal age and BMI, and the gestation the ultrasound was performed at were compared between the male and female cohorts. No significant difference was observed between maternal age, BMI, and percentage of primiparous women in each group. Ethnicity showed an excess of women of “other” ethnicity in the female fetus cohort, but otherwise the groups were not statistically different.

Findings from this study of sex specific variations in Middle cerebral artery Doppler indices and umbilical venous flow have not been previously reported. Although the clinical application of results from this study may be limited at the present time, they do provide further insight into gender specific circulatory differences in the fetal period. Our findings are also intriguing, particularly as more recent data suggest that although these cerebral perfusion differences do not persist into the neonatal period [25],there is evidence that as adults, women have higher cerebral blood flow compared to men [26].
